# Supplementary material for: Resolving the full spectrum of human genome variation using Linked-Reads
Source: Genome Res. 2019 Apr;29(4):635–45. doi: 10.1101/gr.234443.118 (PMC6442396; doi:10.1101/gr.234443.118)
Supplement: Supplemental Material [file supp_gr.234443.118_Supplemental_longranger-2.2.zip › longranger-2.2.tar.bz2!/lr2.2-public/lib/stan/cmdstan/stan/doc/api/html/agrad_2fwd_2matrix_2mdivide__left_8hpp_source.html]

Stan: src/stan/agrad/fwd/matrix/mdivide\_left.hpp Source File


|  |  |
| --- | --- |
|  | Stan  2.5.0  probability, sampling & optimization |


- Main Page
- Related Pages
- Namespaces
- Classes
- Files

- File List
- File Members

All Classes Namespaces Files Functions Variables Typedefs Enumerations Enumerator Friends Macros Pages

- src
- stan
- agrad
- fwd
- matrix

mdivide\_left.hpp

Go to the documentation of this file.

1 #ifndef STAN\_\_AGRAD\_\_FWD\_\_MATRIX\_\_COLUMNS\_MDIVIDE\_LEFT\_HPP

2 #define STAN\_\_AGRAD\_\_FWD\_\_MATRIX\_\_COLUMNS\_MDIVIDE\_LEFT\_HPP

3

4 #include <vector>

5 #include <stan/math/matrix/Eigen.hpp>

6 #include <stan/math/matrix/typedefs.hpp>

7 #include <stan/math/error\_handling/matrix/check\_multiplicable.hpp>

8 #include <stan/agrad/fwd/fvar.hpp>

9 #include <stan/agrad/fwd/matrix/typedefs.hpp>

10 #include <stan/agrad/fwd/matrix/inverse.hpp>

11 #include <stan/agrad/fwd/matrix/multiply.hpp>

12 #include <stan/math/matrix/multiply.hpp>

13 #include <stan/agrad/fwd/matrix/to\_fvar.hpp>

14 #include <stan/math/matrix/inverse.hpp>

15 #include <stan/agrad/fwd/matrix/inverse.hpp>

16 #include <stan/math/matrix/mdivide\_left.hpp>

17 #include <stan/math/error\_handling/matrix/check\_square.hpp>

18

19 namespace stan {

20  namespace agrad {

21

22  template <typename T, int R1,int C1,int R2,int C2>

23  inline

24  Eigen::Matrix<fvar<T>,R1,C2>

25  mdivide\_left(const Eigen::Matrix<fvar<T>,R1,C1> &A,

26  const Eigen::Matrix<fvar<T>,R2,C2> &b) {

27

28  using stan::math::multiply;

29  using stan::math::mdivide\_left;

30  stan::math::check\_square("mdivide\_left(%1%)",A,"A",(double\*)0);

31  stan::math::check\_multiplicable("mdivide\_left(%1%)",A,"A",

32  b,"b",(double\*)0);

33

34  Eigen::Matrix<T,R1,C2> inv\_A\_mult\_b(A.rows(),b.cols());

35  Eigen::Matrix<T,R1,C2> inv\_A\_mult\_deriv\_b(A.rows(),b.cols());

36  Eigen::Matrix<T,R1,C1> inv\_A\_mult\_deriv\_A(A.rows(),A.cols());

37  Eigen::Matrix<T,R1,C1> val\_A(A.rows(),A.cols());

38  Eigen::Matrix<T,R1,C1> deriv\_A(A.rows(),A.cols());

39  Eigen::Matrix<T,R2,C2> val\_b(b.rows(),b.cols());

40  Eigen::Matrix<T,R2,C2> deriv\_b(b.rows(),b.cols());

41

42  for (int j = 0; j < A.cols(); j++) {

43  for(int i = 0; i < A.rows(); i++) {

44  val\_A(i,j) = A(i,j).val\_;

45  deriv\_A(i,j) = A(i,j).d\_;

46  }

47  }

48

49  for (int j = 0; j < b.cols(); j++) {

50  for(int i = 0; i < b.rows(); i++) {

51  val\_b(i,j) = b(i,j).val\_;

52  deriv\_b(i,j) = b(i,j).d\_;

53  }

54  }

55

56  inv\_A\_mult\_b = mdivide\_left(val\_A, val\_b);

57  inv\_A\_mult\_deriv\_b = mdivide\_left(val\_A, deriv\_b);

58  inv\_A\_mult\_deriv\_A = mdivide\_left(val\_A, deriv\_A);

59

60  Eigen::Matrix<T,R1,C2> deriv(A.rows(), b.cols());

61  deriv = inv\_A\_mult\_deriv\_b - multiply(inv\_A\_mult\_deriv\_A, inv\_A\_mult\_b);

62

63  return stan::agrad::to\_fvar(inv\_A\_mult\_b, deriv);

64  }

65

66  template <typename T, int R1,int C1,int R2,int C2>

67  inline

68  Eigen::Matrix<fvar<T>,R1,C2>

69  mdivide\_left(const Eigen::Matrix<double,R1,C1> &A,

70  const Eigen::Matrix<fvar<T>,R2,C2> &b) {

71

72  using stan::math::multiply;

73  using stan::math::mdivide\_left;

74  stan::math::check\_square("mdivide\_left(%1%)",A,"A",(double\*)0);

75  stan::math::check\_multiplicable("mdivide\_left(%1%)",A,"A",

76  b,"b",(double\*)0);

77

78  Eigen::Matrix<T,R2,C2> val\_b(b.rows(),b.cols());

79  Eigen::Matrix<T,R2,C2> deriv\_b(b.rows(),b.cols());

80

81  for (int j = 0; j < b.cols(); j++) {

82  for(int i = 0; i < b.rows(); i++) {

83  val\_b(i,j) = b(i,j).val\_;

84  deriv\_b(i,j) = b(i,j).d\_;

85  }

86  }

87

88  return stan::agrad::to\_fvar(mdivide\_left(A, val\_b),

89  mdivide\_left(A, deriv\_b));

90  }

91

92  template <typename T, int R1,int C1,int R2,int C2>

93  inline

94  Eigen::Matrix<fvar<T>,R1,C2>

95  mdivide\_left(const Eigen::Matrix<fvar<T>,R1,C1> &A,

96  const Eigen::Matrix<double,R2,C2> &b) {

97

98  using stan::math::multiply;

99  using stan::math::mdivide\_left;

100  stan::math::check\_square("mdivide\_left(%1%)",A,"A",(double\*)0);

101  stan::math::check\_multiplicable("mdivide\_left(%1%)",A,"A",

102  b,"b",(double\*)0);

103

104  Eigen::Matrix<T,R1,C2>

105  inv\_A\_mult\_b(A.rows(),b.cols());

106  Eigen::Matrix<T,R1,C1> inv\_A\_mult\_deriv\_A(A.rows(),A.cols());

107  Eigen::Matrix<T,R1,C1> val\_A(A.rows(),A.cols());

108  Eigen::Matrix<T,R1,C1> deriv\_A(A.rows(),A.cols());

109

110  for (int j = 0; j < A.cols(); j++) {

111  for(int i = 0; i < A.rows(); i++) {

112  val\_A(i,j) = A(i,j).val\_;

113  deriv\_A(i,j) = A(i,j).d\_;

114  }

115  }

116

117  inv\_A\_mult\_b = mdivide\_left(val\_A, b);

118  inv\_A\_mult\_deriv\_A = mdivide\_left(val\_A, deriv\_A);

119

120  Eigen::Matrix<T,R1,C2> deriv(A.rows(), b.cols());

121  deriv = -multiply(inv\_A\_mult\_deriv\_A, inv\_A\_mult\_b);

122

123  return stan::agrad::to\_fvar(inv\_A\_mult\_b, deriv);

124  }

125  }

126 }

127 #endif

check\_multiplicable.hpp

inverse.hpp

typedefs.hpp

stan::agrad::mdivide\_left

Eigen::Matrix< fvar< T >, R1, C2 > mdivide\_left(const Eigen::Matrix< fvar< T >, R1, C1 > &A, const Eigen::Matrix< fvar< T >, R2, C2 > &b)

**Definition:** mdivide\_left.hpp:25

stan::agrad::fvar

**Definition:** fvar.hpp:13

stan::agrad::to\_fvar

fvar< T > to\_fvar(const T &x)

**Definition:** to\_fvar.hpp:17

typedefs.hpp

stan::agrad::multiply

Eigen::Matrix< fvar< T >, R1, C1 > multiply(const Eigen::Matrix< fvar< T >, R1, C1 > &m, const fvar< T > &c)

**Definition:** multiply.hpp:21

stan::math::check\_multiplicable

bool check\_multiplicable(const char \*function, const T1 &y1, const char \*name1, const T2 &y2, const char \*name2, T\_result \*result)

**Definition:** check\_multiplicable.hpp:15

fvar.hpp

inverse.hpp

Eigen.hpp

to\_fvar.hpp

mdivide\_left.hpp

multiply.hpp

stan::math::multiply

boost::enable\_if\_c< boost::is\_arithmetic< T >::value, Eigen::Matrix< double, R, C > >::type multiply(const Eigen::Matrix< double, R, C > &m, T c)

Return specified matrix multiplied by specified scalar.

**Definition:** multiply.hpp:25

multiply.hpp

stan::math::check\_square

bool check\_square(const char \*function, const Eigen::Matrix< T\_y, Eigen::Dynamic, Eigen::Dynamic > &y, const char \*name, T\_result \*result)

Return true if the specified matrix is square.

**Definition:** check\_square.hpp:24

stan::math::mdivide\_left

Eigen::Matrix< typename boost::math::tools::promote\_args< T1, T2 >::type, R1, C2 > mdivide\_left(const Eigen::Matrix< T1, R1, C1 > &A, const Eigen::Matrix< T2, R2, C2 > &b)

Returns the solution of the system Ax=b.

**Definition:** mdivide\_left.hpp:24

check\_square.hpp


---

[ Stan Home Page ]

*© 2011–2014,
Stan Development Team.*
